# Surgical trends in elderly patients with proximal humeral fractures in South Korea: a population-based study

**DOI:** 10.1186/s12891-019-2515-2

**Published:** 2019-03-30

**Authors:** Young-Hoon Jo, Kwang-Hyun Lee, Bong-Gun Lee

**Affiliations:** 1 Department of Orthopaedic Surgery, Sungmin Hospital, 70 Sinseok-ro, Seo-gu, Incheon, 22789 Republic of Korea; 2 Department of Orthopaedic Surgery, Hanyang University College of Medicine, 222 Wangsimni-ro, Seongdong-gu, Seoul 04763 Republic of Korea

**Keywords:** Proximal humeral fracture, Epidemiology, Surgery, Trend

## Abstract

**Background:**

It is known that there are large regional variations in treatment methods for the management of proximal humeral fractures. The objective of this study was to investigate the national surgical trends in elderly patients with proximal humeral fractures in South Korea.

**Methods:**

We analyzed the Korean Health Insurance Review and Assessment Service database from 2008 to 2016. International Classification of Diseases, 10th revision codes and procedure codes were used to identify patients aged ≥65 years with proximal humeral fractures.

**Results:**

A total of 69,120 proximal humeral fractures were identified from 2008 to 2016. The overall operative rate for proximal humeral fractures increased steadily from 24.6% in 2008 to 36.8% in 2016 (*p* < 0.001). The rate of cases treated with open reduction and internal fixation tended to increase each year, from 71.5% of the overall surgeries in 2008 to 85.6% in 2016; conversely, the rate of cases treated with closed reduction and internal fixation tended to decrease from 19.9% in 2008 to 4.5% in 2016. In terms of type of arthroplasty procedure, the rate of cases treated with reverse shoulder arthroplasty tended to increase significantly each year, from 8.2% of the overall arthroplasty procedures in 2008 to 52.0% in 2016 (*p* < 0.001). The proportion of reverse shoulder arthroplasty was shown to increase especially in patients aged 80 years or older.

**Conclusion:**

Overall, our findings indicated that surgical treatment of proximal humeral fractures, particularly by open reduction and internal fixation, continues to increase; in terms of type of arthroplasty procedure, the rate of cases treated with reverse shoulder arthroplasty tended to increase.

## Background

Proximal humeral fractures (PHFs) account for approximately 5% of all fractures, and are the third most common fractures in patients older than 65 years following hip and distal radius fractures [1, 2]. Although nondisplaced or minimally displaced PHFs can be treated nonoperatively, displaced or unstable PHFs often require surgical treatment [3, 4]. The proportion of cases with PHFs undergoing surgical treatment has been growing over recent years, and in New York State approximately 30% of PHFs were treated surgically in 2010 [5–7].

**Fig. 1:**
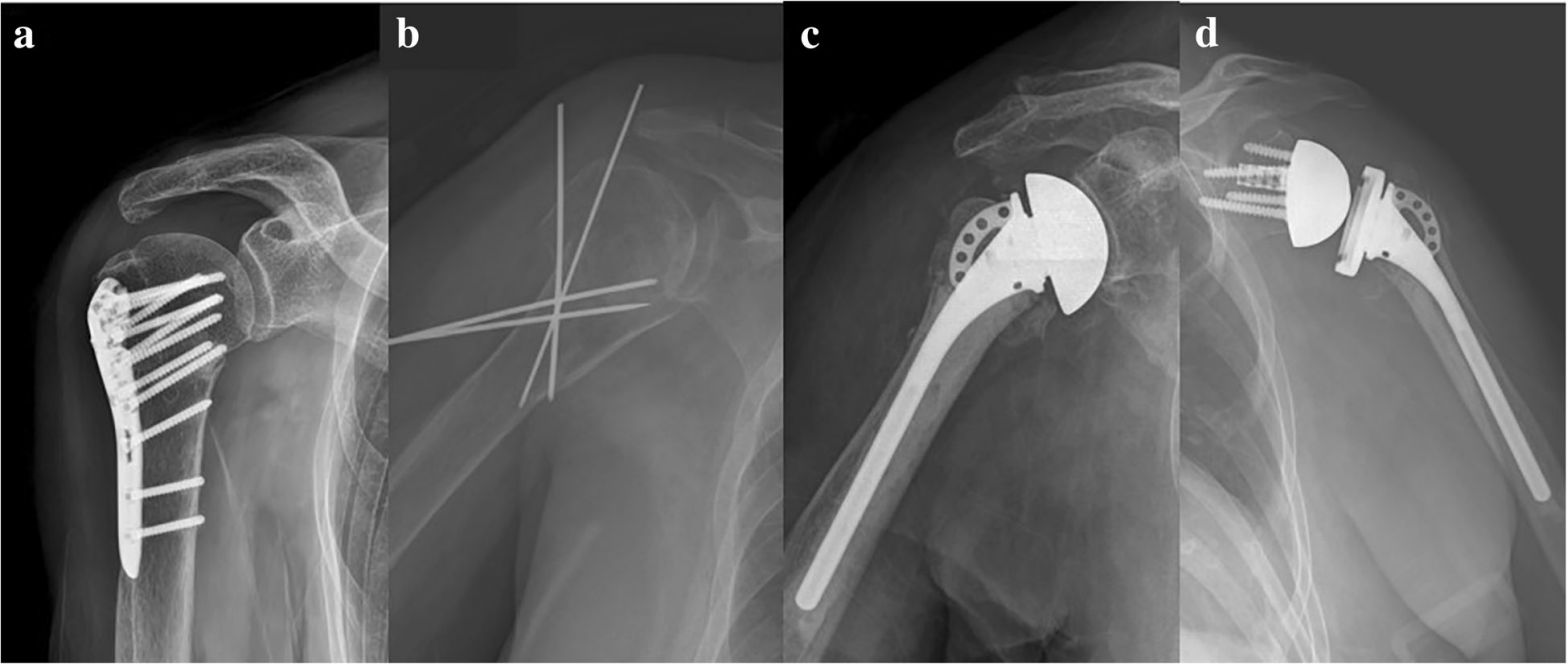
Plain radiographs demonstrating types of surgical treatment of proximal humeral fractures: (**a**) open reduction and internal fixation (**b**) closed reduction and internal fixation (**c**) hemiarthroplasty (**d**) reverse shoulder arthroplasty. All surgeries took place at Hanyang University Medical Centre, and patients gave informed consent for the publication of their radiographs

Surgical treatments for PHFs include open reduction and internal fixation (ORIF), closed reduction and internal fixation (CRIF), hemiarthroplasty (HA), and reverse shoulder arthroplasty (RSA) (Fig. 1) [8]. Since the development of the biomechanically more advantageous locking plates and locking intramedullary nails, the surgical indications for ORIF have been extended to elderly patients with osteoporosis [9, 10]. However, for both devices, high implant-related complication rates and reoperation rates have been reported [11–13]. Although HA may be an attractive treatment method for comminuted fractures in elderly patients, it is known to have poor outcomes when nonunion or malunion of the tuberosity occurs [14, 15]. RSA has recently been used to treat PHFs in elderly patients and reported to produce satisfactory outcomes regardless of whether there is union of the tuberosity [16, 17]. However, there is still no clear evidence or consensus regarding the most appropriate method of surgery for different fracture types [18, 19]. Therefore, it is known that there are large regional variations in the methods used for treatment [7, 8]. To the best of our knowledge, there has not been any report on the treatment trends for PHFs in Asian populations.

Thus, the objective of this study was to investigate national surgical trends in elderly patients aged ≥65 years with PHFs in South Korea based on an analysis of nationwide data acquired from the Korean Health Insurance Review and Assessment Service (HIRA) database. Our hypotheses were as follows: first, the proportion of surgical treatments for PHFs would increase; and second, the use of HA would decrease while that of RSA would increase in the treatment of PHFs.

## Methods

### Data source

The authors analysed nationwide data from 2008 to 2016 obtained from the HIRA database. In South Korea, the National Health Insurance covers 100% of the population, including 97% of health insurance and 3% of medical aid [20]. All healthcare providers submit claims data for inpatient and outpatient management, including diagnostic codes (classified according to the International Classification of Diseases, 10th revision [ICD-10]), procedure codes, and demographic information, to the HIRA to request reimbursement for medical costs from the National Health Insurance service. Hence, medical records of almost all outpatients or hospitalised patients at health-care institutions in South Korea are prospectively recorded in the HIRA database.

### Data collection

We conducted a survey of patients aged ≥65 years with newly diagnosed PHFs in South Korea between 2008 and 2016 [7, 21, 22]. First, patients with PHFs who received surgical treatment were identified with the ICD-10 codes (S42.2) and the operation codes (ORIF: N0602 and N0612; CRIF: N0992; HA: N2711 and N2716; RSA: N2071 and N2076) from the HIRA database (Table 1) [23]. The operations codes for ORIF include osteosynthesis by intramedullary nailing. Subsequently, patients who underwent conservative treatment for PHFs were identified as those for whom ICD-10 codes (S42.2) codes were entered, but operation codes were not. The HIRA data for 2007 was required to ensure that the observed PHFs were not recurrent entries of fractures that had occurred before 2008 [7]. To avoid multiple counting of ICD-10 codes for PHFs that were conservatively treated more than once during the study period, only the data obtained when the ICD-10 code was first entered for each patient were included [21].

**Table 1:** ICD-10 diagnosis codes and procedure codes of proximal humeral fractures

	Code	Description
ICD-10 code^a^	S42.2	Fracture of upper end of humerus
Operation code in South Korea	N0602 or N0612	ORIF^b^ for humerus or scapula
	N0992	Closed pinning for humerus or scapula
	N2711 or N2716	Replacement hemiarthroplasty-shoulder
	N2071 or N2076	Replacement total arthroplasty-shoulder

*ICD-10*^a^ International Classification of Diseases, 10th revision, *ORIF*^b^ open reduction with internal fixation

We examined patient data to identify the year of fracture occurrence, age at which it occurred, sex of patients, whether surgery was performed, operation code, and inpatient cost. Primary total shoulder arthroplasty is rarely performed for PHFs, as the glenoid is typically uninvolved. Therefore, the patients for whom operation codes for total shoulder arthroplasty for PHF (N2071 and N2076) were entered were assumed to have undergone RSA [6]. The surgical treatments for PHFs included ORIF (N0602 and N0612), CRIF (N0992), HA (N2711 and N2716), and RSA (N2071 and N2076) [8]. HA and RSA were classified as arthroplasty procedures. We investigated the incidence of PHFs by year, cost of surgery, and type of surgery to analyze surgical trends.

### Statistical analysis

We calculated age-adjusted and sex-specific incidence rates per 100,000 persons with PHFs using the 2010 United States population as the standard population [24]. Estimated year-specific, age-specific, and sex-specific populations were obtained from the Statistics Korea website (http://www.kosis.kr). The annual percentage changes in the age-adjusted incidence rates of PHFs were calculated from 2008 to 2016 using joinpoint regression analysis (Joinpoint Regression Program, version 4.3.1.0; National Cancer Institute, Bethesda, MD, USA) [20, 21].

All other data sets were analyzed using SAS statistical software version 9.13 (SAS Institute, Cary, NC, USA). We analyzed the correlations between time and the number of PHFs by using a simple linear regression analysis [8]. We used the Cochran-Armitage trend test to analyze the changes in the proportion of surgical treatments, arthroplasty procedures, and RSA procedures over time. Proportions for surgical management of PHFs by age group in 2008 and 2016 was compared with use of Pearson chi-square tests [7]. Values of *p* < 0.05 were considered significant.

## Results

A total of 69,120 patients aged ≥65 years with PHFs were identified from 2008 to 2016 in South Korea. Of these, 14,734 and 54,386 cases involved male and female patients, respectively; female patients accounted for 78.7% of the overall cohort. The mean age of patients with PHFs was 76.3 (± 7.2) years. The number of cases of PHF per year increased significantly from 6357 in 2008 to 8919 in 2016 (*r* = 0.978, *p* < 0.001) (Table 2). However, the age-adjusted incidence rate per 100,000 persons decreased slightly from 147.5 in 2008 to 140.0 in 2016 (Table 2). The annual percentage change in the age-adjusted incidence rate for study period was calculated as − 0.7% (95% confidence interval [CI], − 1.5-0.1%), which was not statistically significant (*p* = 0.08).

**Table 2:** Number of cases and age-adjusted rates of proximal humeral fractures of patients ≥65 years from 2008 to 2016

Years	No. of cases			Age adjusted incidence rates^a^		
	Total	Men	Women	Total	Men	Women
2008	6357	1392	4965	147.5	77.8	184.2
2009	6549	1344	5205	142.0	71.4	181.2
2010	7443	1577	5866	154.8	80.7	196.1
2011	7301	1532	5769	145.9	75.9	185.7
2012	7814	1691	6123	147.6	79.5	187.1
2013	7795	1653	6142	141.5	76.1	180.2
2014	8255	1783	6472	141.7	78.2	180.8
2015	8687	1887	6800	143.1	79.0	182.6
2016	8919	1875	7044	140.0	73.2	182.0
Overall	69,120	14,734	54,386			

^a^ Use of the United States population in 2010 as the control

**Fig. 2:**
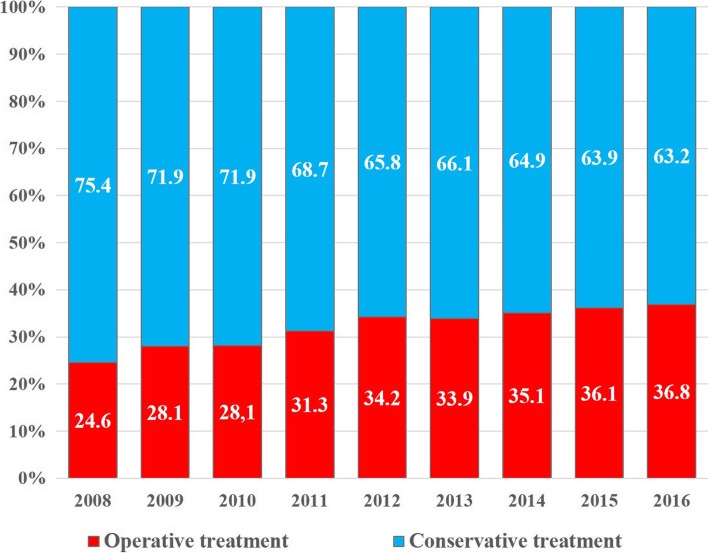
Proportions of surgical treatment in patients ≥65 years of age with proximal humeral fractures, by year. The values are percentage values

**Fig. 3:**
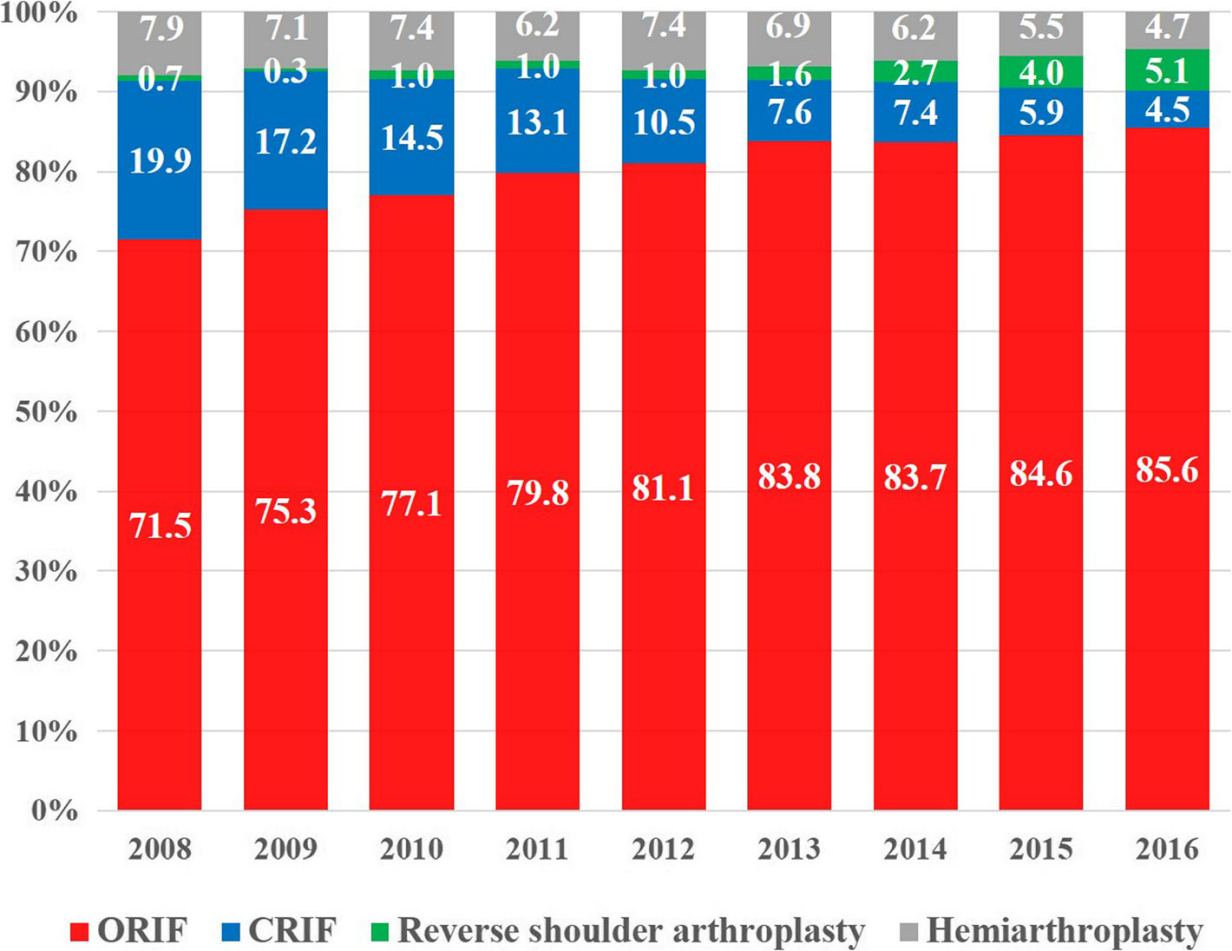
Proportions of operation type in patients ≥65 years of age with proximal humeral fractures, by year**.** The values are percentage values

Conservative treatment was the most common treatment method (67.6%) followed by ORIF (26.2%), CRIF (3.3%), HA (2.1%), and RSA (0.7%). The overall operative rate for PHFs increased steadily from 24.6% in 2008 to 36.8% in 2016 (Fig. 2, *p* < 0.001). The rate of cases treated with ORIF tended to increase each year, from 71.5% of the overall surgeries in 2008 to 85.6% in 2016, while the rate of CRIF decreased from 19.9% in 2008 to 4.5% in 2016 (Fig. 3).

**Fig. 4:**
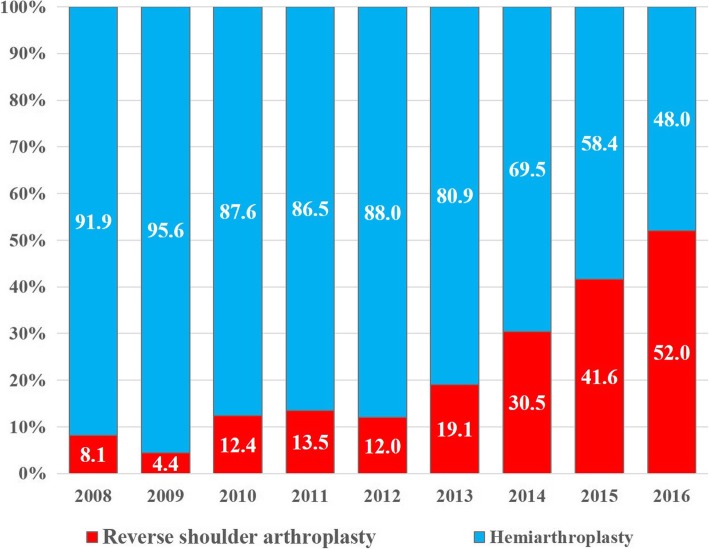
The ratios of reverse shoulder arthroplasty and hemiarthroplasty in patients ≥65 years of age who underwent arthroplasty procedures for PHFs, by year. The values are percentage values

The rate of cases involving arthroplasty procedures increased slightly, from 8.6% of cases of operative PHFs in 2008 to 9.9% in 2016 (*p* < 0.001). In terms of type of arthroplasty procedure, the rate of cases treated with RSA tended to increase significantly each year, from 8.1% of the overall arthroplasty procedures performed in 2008 to 52.0% in 2016, while the rate of cases treated using HA decreased (Fig. 4, *p* < 0.001). The proportion of RSA of the overall procedures has shown to increase especially significant in patients aged 80 years or older (Table 3). In this population, the proportion of use of RSA increased dramatically, from 1% of the overall procedures in 2008 to 9% in 2016 (*p* < 0.001).

**Table 3:** Proportions for surgical management of proximal humeral fractures by age group in 2008 and 2016

Years	Patients aged 65 to 79				Patients older than 80				*p*-value^a^
	ORIF (%)	CRIF (%)	HA (%)	RSA (%)	ORIF (%)	CRIF (%)	HA (%)	RSA (%)	
2008	73.4	18.4	7.6	0.6	65.9	24.1	9.0	1.0	0.036
2016	88.4	4.0	4.3	3.3	79.5	5.7	5.8	9.0	< 0.001
*p*-value	< 0.001				< 0.001				

^a^*p* values were estimated by comparing between age groups in the same year

The mean hospitalization cost was the highest for RSA (7017 USD), followed by HA (6124 USD), ORIF (3454 USD), and CRIF (2280 USD). The mean hospitalization cost for patients who underwent surgical treatment for PHFs significantly increased from 3244 USD in 2008 to 4125 USD in 2016 (*r* = 0.907, *p* < 0.001).

## Discussion

In this study, we used nationwide data to analyse surgical trends among patients aged 65 years or older with PHFs in South Korea. There were no significant changes in the age-adjusted incidence rates of PHF during the study period, and the proportion of surgical treatments increased annually. Among the different surgical methods, the proportion of use of ORIF gradually increased up to 85.6% of the overall procedures performed in 2016. Among arthroplasty procedures, the proportion of use of RSA increased annually whereas that of HA decreased; this trend was more evident among patients aged 80 years or older.

Although the number of cases of PHF per year significantly increased from 2008 to 2016, there were no significant changes in the age-adjusted incidence rates of PHFs. This may be because the incidence rates were calculated among the parent population, which consisted of persons aged 65 years or older; therefore, although the number of cases of PHF increased, the parent population also increased due to population aging. This resulted in no significant change in the incidence rates. In fact, according to the Statistics Korea website, the estimated total population size of persons aged 65 years or older significantly increased from 4,988,592 in 2008 to 6,762,842 in 2016. If the incidence rate of PHFs had been calculated in all age groups without age-adjusted in this study, it would have been expected to increase steadily during the study period as population aging continued [25].

Excellent outcomes can be obtained for nondisplaced or minimally displaced PHFs using conservative treatment [26]. There is insufficient evidence that surgical treatments produce better outcomes for displaced PHFs than conservative treatments [27, 28]. However, in this study, the proportion of use of surgical treatments was observed to gradually increase from 24.6% in 2008 to 36.8% in 2016. In other population-based studies, the proportion of surgical treatments for PHFs was also observed to increase over the years [5, 8]. It appears that the preference for surgical treatments increased due to various factors such as the development of new surgical techniques and instruments, better understanding about the importance of accurate reduction of the greater tuberosity, and an increased number of surgeons who can surgically treat PHFs [7, 20, 23, 29].

The rate of use of ORIF consistently increased during the study period, whereas that of CRIF decreased. Huttunen et al. [23] reported that the rate of ORIF using plates increased two-fold from 2002, when locking plates were introduced, to 2009. Locking plates produce greater angular stability and screw anchorage in osteoporotic bones [30], and thus, have stronger holding power on the humeral head than conventional plates [31]. In addition, the intramedullary nail can be also a useful tool for PHF fixation; its minimally invasive approach, secure tuberosity-specific locking screws, and ability for early rehabilitation often yields satisfactory clinical outcomes [10, 12]. Although CRIF has the advantage of less soft tissue injuries, the rate of reduction loss following CRIF has been reported to be up to 27%, and the rate of fixation failure is higher in elderly patients with osteoporosis [32]. It appears that the rate of use of ORIF has increased because many surgeons in South Korea prefer anatomic reduction and rigid fixation for early range of motion exercise.

Elderly patients with 3- and 4-part PHFs have increased rates of avascular necrosis of the humeral head and increased complication rates following ORIF [12, 33, 34]. Therefore, arthroplasty procedures may be a good alternative for these fracture types. In this study, the proportion of use of RSA increased whereas that of HA decreased among the arthroplasty procedures used. HA is known to have poor functional outcomes in elderly patients when nonunion or malunion of the tuberosity occurs [14, 15]. The rate of nonunion of the tuberosity has been reported to be as high as 65% [16, 35]. Conversely, RSA has excellent functional outcomes regardless of tuberosity union or malunion [16, 17], and according to other population-based studies, the preference for RSA has been increasing [8, 36]. The preference for RSA has also been increasing in South Korea; RSA was preferred over HA in 2016.

The preference for RSA over HA was especially high among patients aged 80 years or older. This may be because of the higher prevalence rotator cuff disease among older patients [37] and the criteria of insurance coverage for shoulder arthroplasty in South Korea. Before October 2017, only elderly patients aged ≥80 years with 3- or 4-part PHFs received insurance coverage for RSA in South Korea. This system was a major constraint for surgeons to choose the surgical treatment of patients aged < 80 with PHFs in South Korea. However, after October 2017, the criteria of insurance coverage for RSA was changed to include patients aged 70 years or older. Thus, the preference for RSA is expected to increase in younger age groups.

The mean hospitalization cost for patients who underwent surgical treatment for PHFs significantly increased from 2008 to 2016. One reason for this increase in cost may be the high costs of implants for ORIF and RSA, the preference for which has increased. To accurately analyze the cost-effectiveness of surgical methods, costs for reoperation, improving quality of life, and long-term patient satisfaction must be considered in addition to hospitalization costs. Additional research is needed to comprehensively analyze these factors.

Although this study employed a large sample size based on a nationwide database, it also had some limitations. First, we only included the data obtained when the ICD-10 code was first entered for each patient. Although we could avoid multiple counting using this method, PHFs that occurred twice or more in a single patient during the study period could be measured as a single PHF, resulting in the underestimation of the number of PHFs. Second, there is no separate operation code for RSA in South Korea. In South Korea, the same operation code is registered for total shoulder arthroplasty and RSA (Table 1). However, we believe that total shoulder arthroplasty is rarely performed for PHFs [6]. Third, because the HIRA data provided no information on clinical outcomes, the clinical outcomes between operative procedures were not comparable in this study. Fourth, the insurance coverage served as a bias that could have caused high preference for RSA over HA in only patients over 80 years of age. If there had been no insurance bias, the RSA preference might have been higher than HA even for those under 80 years of age in 2016. Finally, there are possibilities of some code errors in large databases.

## Conclusions

In South Korea, the proportion of use of surgical treatments for PHFs tended to increase. In addition, ORIF had the highest proportion among surgical treatments, and this value increased every year. On the other hand, the use of CRIF for treatment of PHFs decreased. Among arthroplasty procedures, the proportion of use of RSA tended to increase; higher preference for RSA over HA was observed in 2016.

## Abbreviations

CRIF: Close reduction and internal fixation
HA: Hemiarthroplasty
HIRA: Health Insurance Review and Assessment Service
ICD-10: International Classification of Diseases, 10th revision
ORIF: Open reduction and internal fixation
PHFs: Proximal humeral fractures
RSA: Reverse shoulder arthroplasty

## References

[1] Court-Brown CM, Garg A, McQueen MM (2001). The epidemiology of proximal humeral fractures. Acta Orthop Scand.

[2] Baron JA, Karagas M, Barrett J, Kniffin W, Malenka D, Mayor M (1996). Basic epidemiology of fractures of the upper and lower limb among Americans over 65 years of age. Epidemiology..

[3] Robinson CM, Amin AK, Godley KC, Murray IR, White TO (2011). Modern perspectives of open reduction and plate fixation of proximal humerus fractures. J Orthop Trauma.

[4] Kleinlugtenbelt YV, Bhandari M (2015). Cochrane in CORR®: interventions for treating proximal humeral fractures in adults (review). Clin Orthop Relat Res.

[5] Khatib O, Onyekwelu I, Zuckerman JD (2014). The incidence of proximal humeral fractures in New York state from 1990 through 2010 with an emphasis on operative management in patients aged 65 years or older. J Shoulder Elb Surg.

[6] Hasty EK, Jernigan EW, Soo A, Varkey DT, Kamath GV (2017). Trends in surgical management and costs for operative treatment of proximal Humerus fractures in the elderly. Orthopedics..

[7] Bell JE, Leung BC, Spratt KF, Koval KJ, Weinstein JD, Goodman DC (2011). Trends and variation in incidence, surgical treatment, and repeat surgery of proximal humeral fractures in the elderly. J Bone Joint Surg Am.

[8] Sabesan VJ, Lombardo D, Petersen-Fitts G, Weisman M, Ramthun K, Whaley J (2017). National trends in proximal humerus fracture treatment patterns. Aging Clin Exp Res.

[9] Agudelo J, Schurmann M, Stahel P, Helwig P, Morgan SJ, Zechel W (2007). Analysis of efficacy and failure in proximal humerus fractures treated with locking plates. J Orthop Trauma.

[10] Gracitelli MEC, Malavolta EA, Assuncao JH, Ferreira Neto AA, Silva JS, Hernandez AJ (2017). Locking intramedullary nails versus locking plates for the treatment of proximal humerus fractures. Expert Rev Med Devices.

[11] Sudkamp N, Bayer J, Hepp P, Voigt C, Oestern H, Kaab M (2009). Open reduction and internal fixation of proximal humeral fractures with use of the locking proximal humerus plate. Results of a prospective, multicenter, observational study. J Bone Joint Surg Am.

[12] Konrad G, Audige L, Lambert S, Hertel R, Sudkamp NP (2012). Similar outcomes for nail versus plate fixation of three-part proximal humeral fractures. Clin Orthop Relat Res.

[13] Gracitelli ME, Malavolta EA, Assuncao JH, Kojima KE, dos Reis PR, Silva JS (2016). Locking intramedullary nails compared with locking plates for two- and three-part proximal humeral surgical neck fractures: a randomized controlled trial. J Shoulder Elb Surg.

[14] Boileau P, Krishnan SG, Tinsi L, Walch G, Coste JS, Mole D (2002). Tuberosity malposition and migration: reasons for poor outcomes after hemiarthroplasty for displaced fractures of the proximal humerus. J Shoulder Elb Surg.

[15] Antuña SA, Sperling JW, Cofield RH (2008). Shoulder hemiarthroplasty for acute fractures of the proximal humerus: a minimum five-year follow-up. J Shoulder Elb Surg.

[16] Cuff DJ, Pupello DR (2013). Comparison of hemiarthroplasty and reverse shoulder arthroplasty for the treatment of proximal humeral fractures in elderly patients. J Bone Joint Surg Am.

[17] Chun YM, Kim DS, Lee DH, Shin SJ (2017). Reverse shoulder arthroplasty for four-part proximal humerus fracture in elderly patients: can a healed tuberosity improve the functional outcomes?. J Shoulder Elb Surg.

[18] Handoll HH, Ollivere BJ, Rollins KE (2012). Interventions for treating proximal humeral fractures in adults. Cochrane Database Syst Rev.

[19] Handoll HH, Brorson S (2015). Interventions for treating proximal humeral fractures in adults. Cochrane Database Syst Rev.

[20] Jo YH, Lee KH, Kim SJ, Kim J, Lee BG (2017). National trends in surgery for rotator cuff disease in Korea. J Korean Med Sci.

[21] Martinez-Huedo MA, Jimenez-Garcia R, Mora-Zamorano E, Hernandez-Barrera V, Villanueva-Martinez M (2017). Trends in incidence of proximal humerus fractures, surgical procedures and outcomes among elderly hospitalized patients with and without type 2 diabetes in Spain (2001-2013). BMC Musculoskelet Disord.

[22] Rajaee SS, Yalamanchili D, Noori N, Debbi E, Mirocha J, Lin CA (2017). Increasing use of reverse total shoulder arthroplasty for proximal humerus fractures in elderly patients. Orthopedics..

[23] Huttunen TT, Launonen AP, Pihlajamaki H, Kannus P, Mattila VM (2012). Trends in the surgical treatment of proximal humeral fractures - a nationwide 23-year study in Finland. BMC Musculoskelet Disord.

[24] Jo YH, Lee BG, Kim HS, Kim JH, Lee CH, Kim SJ (2018). Incidence and seasonal variation of distal radius fractures in Korea: a population-based study. J Korean Med Sci.

[25] Muramatsu N, Akiyama H (2011). Japan: super-aging society preparing for the future. Gerontologist..

[26] Koval KJ, Gallagher MA, Marsicano JG, Cuomo F, McShinawy A, Zuckerman JD (1997). Functional outcome after minimally displaced fractures of the proximal part of the humerus. J Bone Joint Surg Am.

[27] Rangan A, Handoll H, Brealey S, Jefferson L, Keding A, Martin BC (2015). Surgical vs nonsurgical treatment of adults with displaced fractures of the proximal humerus: the PROFHER randomized clinical trial. JAMA..

[28] Rabi S, Evaniew N, Sprague SA, Bhandari M, Slobogean GP (2015). Operative vs non-operative management of displaced proximal humeral fractures in the elderly: a systematic review and meta-analysis of randomized controlled trials. World J Orthop.

[29] Bono CM, Renard R, Levine RG, Levy AS (2001). Effect of displacement of fractures of the greater tuberosity on the mechanics of the shoulder. J Bone Joint Surg Br.

[30] Karataglis D, Stavridis SI, Petsatodis G, Papadopoulos P, Christodoulou A (2011). New trends in fixation of proximal humeral fractures: a review. Injury..

[31] Walsh S, Reindl R, Harvey E, Berry G, Beckman L, Steffen T (2006). Biomechanical comparison of a unique locking plate versus a standard plate for internal fixation of proximal humerus fractures in a cadaveric model. Clin Biomech (Bristol, Avon).

[32] Fenichel I, Oran A, Burstein G, Perry Pritsch M (2006). Percutaneous pinning using threaded pins as a treatment option for unstable two- and three-part fractures of the proximal humerus: a retrospective study. Int Orthop.

[33] Greiner S, Kaab MJ, Haas NP, Bail HJ (2009). Humeral head necrosis rate at mid-term follow-up after open reduction and angular stable plate fixation for proximal humeral fractures. Injury..

[34] Boesmueller S, Wech M, Gregori M, Domaszewski F, Bukaty A, Fialka C (2016). Risk factors for humeral head necrosis and non-union after plating in proximal humeral fractures. Injury..

[35] Reuther F, Muhlhausler B, Wahl D, Nijs S (2010). Functional outcome of shoulder hemiarthroplasty for fractures: a multicentre analysis. Injury..

[36] Rosas S, Law TY, Kurowicki J, Formaini N, Kalandiak SP, Levy JC (2016). Trends in surgical management of proximal humeral fractures in the Medicare population: a nationwide study of records from 2009 to 2012. J Shoulder Elb Surg.

[37] Yamamoto A, Takagishi K, Osawa T, Yanagawa T, Nakajima D, Shitara H (2010). Prevalence and risk factors of a rotator cuff tear in the general population. J Shoulder Elb Surg.

